# TLR4 response mediates ethanol-induced neurodevelopment alterations in a model of fetal alcohol spectrum disorders

**DOI:** 10.1186/s12974-017-0918-2

**Published:** 2017-07-24

**Authors:** María Pascual, Jorge Montesinos, Sandra Montagud-Romero, Jerónimo Forteza, Marta Rodríguez-Arias, José Miñarro, Consuelo Guerri

**Affiliations:** 1 Department of Molecular and Cellular Pathology of Alcohol, Principe Felipe Research Center, C/Eduardo Primo Yúfera 3, 46012 Valencia, Spain; 2 Department of Psychobiology, Facultad de Psicología, Universitat de Valencia, Valencia, Spain; 3 Instituto Valenciano de Patología, Unidad Mixta de Patología Molecular, Principe Felipe Research Center, Valencia, Spain; 4 Red Temática de Investigación Cooperativa en Salud (RETICS-Trastornos Adictivos), Instituto de Salud Carlos III, MICINN and FEDER, Madrid, Spain; 5 Department of Physiology, School of Medicine, Universitat de Valencia, Valencia, Spain

**Keywords:** Fetal alcohol spectrum disorders, TLR4, Neuroinflammation, Prenatal ethanol exposure, Cerebral cortex, Serum, Amniotic fluid, Behavior impairments

## Abstract

**Background:**

Inflammation during brain development participates in the pathogenesis of early brain injury and cognitive dysfunctions. Prenatal ethanol exposure affects the developing brain and causes neural impairment, cognitive and behavioral effects, collectively known as fetal alcohol spectrum disorders (FASD). Our previous studies demonstrate that ethanol activates the innate immune response and TLR4 receptor and causes neuroinflammation, brain damage, and cognitive defects in the developmental brain stage of adolescents. We hypothesize that by activating the TLR4 response, maternal alcohol consumption during pregnancy triggers the release of cytokines and chemokines in both the maternal sera and brains of fetuses/offspring, which impairs brain ontogeny and causes cognitive dysfunction.

**Methods:**

WT and TLR4-KO female mice treated with or without 10% ethanol in the drinking water during gestation and lactation were used. Cytokine/chemokine levels were determined by ELISA in the amniotic fluid, maternal serum, and cerebral cortex, as well as in the offspring cerebral cortex. Microglial and neuronal markers (evaluated by western blotting), myelin proteins (immunohistochemical and western blotting) and synaptic parameters (western blotting and electron microscopy) were assessed in the cortices of the WT and TLR4-KO pups on PND 0, 20, and 66. Behavioral tests (elevated plus maze and passive avoidance) were performed in the WT and TLR4-KO mice on PND 66 exposed or not to ethanol.

**Results:**

We show that alcohol intake during gestation and lactation increases the levels of several cytokines/chemokines (IL-1β, IL-17, MIP-1α, and fractalkine) in the maternal sera, amniotic fluid, and brains of fetuses and offspring. The upregulation of cytokines/chemokines is associated with an increase in activated microglia markers (CD11b and MHC-II), and with a reduction in some synaptic (synaptotagmin, synapsin IIa) and myelin (MBP, PLP) proteins in the brains of offspring on days 0, 20, and 66 (long-term effects). These changes are associated with long-term behavioral impairments, in the 66-day-old alcohol-exposed pups. TLR4-deficient mice are protected against ethanol-induced cytokine/chemokine production in alcohol-treated dams and offspring, along with synaptic and myelin alterations, and the log-term behavioral dysfunction induced by ethanol in offspring.

**Conclusions:**

These results suggest that the immune system activation, through the TLR4 response, might play an important role in the neurodevelopmental defects in FASD.

**Electronic supplementary material:**

The online version of this article (doi:10.1186/s12974-017-0918-2) contains supplementary material, which is available to authorized users.

## Background

Alcohol consumption during pregnancy causes major deleterious effects on the developing human fetus, which leads to a range of physical and cognitive abnormalities in children that persist into adulthood [1, 2]. The wide array of consequences associated with prenatal ethanol exposure, ranging from neurocognitive deficits to physical malformations, is now termed fetal alcohol spectrum disorder (FASD). One of the most disturbing and lasting effects of prenatal alcohol exposure occurs in the developing brain, in which structural and functional defects, along with a range of neuropsychological and behavioral consequences, are observed in children with FASD [3]. Studies in animal models have confirmed that the brain is one of the most vulnerable organs to ethanol, and that alcohol exposure during brain ontogeny leads to important long-term cognitive and behavioral dysfunction. Experimental studies also show that prenatal alcohol exposure affects all brain development stages, from neurogenesis, gliogenesis, and myelination to different mechanisms which include disrupted cell-cell interactions, altered gene expression, oxidative stress, and growth factor signaling disruptions (see refs., [4, 5]). Recent studies have also shown that exposure to even low/moderate levels of alcohol in early mice embryonic developmental stages (e.g., gastrulation or neurulation) is associated with behavioral deficits in spatial learning, memory disabilities, delayed sensorimotor development, hypoactivity, and increased emotionality in adulthood [6–8].

Evidence from the last decade supports the role of the innate immune response and neuroinflammation in the behavioral and cognitive effects induced by binge drinking in the brain maturation stage of adolescents [9, 10]. Activation of the neuroimmune system and glial cells response via the production of pro-inflammatory molecules has also been reported in animal models of FASD [11–16]. Low/moderate prenatal alcohol exposure also induces microglia activation, along with the production of cytokines, in the fetal rat brain, and triggers long-term cognitive dysfunctions [17]. Postnatal ethanol exposure also triggers astrocyte activation and contributes to neuroinflammation in the developing brain [18].

Inflammation is also recognized as being a critical contributor to both normal development and injury outcome in the immature brain. Perinatal immune activation in the mother and/or fetus adversely affects neurodevelopment and increases the risk of developing psychiatric disorders (see refs., [19, 20]). Pattern recognition receptors, such as toll-like receptors (TLRs) in innate immune cells (microglia, mast cells, and macrophages) are important participants in the early phases of injury during development and in the adult brain [19, 21], and can increase central nervous system (CNS) vulnerability. These receptors sense pathogens (PAMPs or pattern-associated molecular patterns) and damaged cells (DAMPs or damaged-associated molecular patterns), and can induce a fast response by activating different signaling pathways, such as nuclear factor-κB (NF-κB), which leads to the production of cytokines and inflammatory mediators [22]. Under physiological conditions, TLRs play a role during brain development [23], but the excessive activation of these receptors can induce inflammation and damage. For instance, in utero exposure to endotoxin LPS (lipopolysaccharide), the ligand of TLR4, induces white matter injury [24], alters the morphology of the pyramidal neurons of the prefrontal cortex and hippocampus in rats [25]. Knocking out TLRs in mice provides neuroprotection in hypoxia-ischemia [26]. Our previous studies have shown that ethanol is able to activate the TLR4 signaling response in glial cells by triggering the production of inflammatory mediators and/or cytokines and causing gliosis, neuroinflammation, myelin disruption, and neural damage in both adult and adolescent brains [10, 27, 28]. However, whether alcohol-induced TLR4 activation is involved in the actions of ethanol on the developing brain is currently unknown.

Recent studies have demonstrated that alcohol consumption triggers greater peripheral immune activation, along with the upregulation of cytokines and chemokines production in females rather than in males [29]. Considering that immune activation in the mother and/or fetus adversely affects neurodevelopment [20], the aim of this study is to evaluate whether ethanol-induced maternal innate immune activation can adversely affect the developing brain by activating glial cells and cytokines release, and whether these effects are linked with the TLR4 response. Here, we provide evidence that maternal ethanol consumption increases the levels of some cytokines in maternal serum and amniotic fluid, and in the cortices of the 15-day-old fetuses, newborn and 20-day-old pups. This inflammatory environment impairs the expression of cortical myelin and synaptic proteins, and causes long-term cognitive and behavior impairments. Elimination of TLR4 protects against the ethanol-induced maternal immune activation and structural and cognitive effects induced by ethanol exposure during the developing brain, which supports the role of neuroimmune system activation and the TLR4 response in neurodevelopmental and neurobehavioral dysfunctions in FASD.

## Methods

### Animals and ethanol treatment

Female C57BL/6 wild-type (WT) (Harlan Ibérica, Barcelona, Spain) and TLR4 knockout (TLR4-KO) mice (C57BL/6 background, kindly provided by Dr. S. Akira, Osaka University, Suita, Japan), which weighed 23–27 g, were used. Both the WT and TLR4-KO female mice were housed in groups of 3–4 each per cage. They were maintained under controlled light and dark (12/12 h), temperature (23 °C) and humidity (60%) conditions for 1 month. Animals were then divided into different groups; (1) control WT (*n* = 15 dams) and TLR4-KO (*n* = 15 dams) mice, which received solid diet and drinking water ad libitum; (2) ethanol-treated WT (*n* = 15 dams) and TLR4-KO (*n* = 15 dams) mice, which received 10% ethanol solution (v/v) in their drinking water and solid diet, ad libitum, for 2 months before mating, as previously described [30]. During this period, the daily food and liquid intake was similar for the WT and KO mice, and also for the alcohol-treated (per mouse and day, food and fluid intake were 3.26 ± 0.91 g and 3.49 ± 0.5 ml of 10% ethanol in water, respectively) and untreated (food/fluid daily intake per mouse was 3.32 ± 0.81 g and 3.55 ± 0.92 ml of water) groups. The ethanol concentration in drinking water was progressively increased for the first 2 weeks to finally reach 12.8 ± 1.2 g/kg of body weight. The body weight gain at 2 months was similar in the WT and TLR4-KO mice treated with or without ethanol, and no differences in hydration between groups were found following the criteria described by [31] (e.g., loss of body weight, reduction of food intake, listlessness/inactivity, increased “skin tent”, etc.). However, to avoid potential dehydration, a hydrogel was also provided to the ethanol-treated mice. The blood ethanol levels in the dark cycle in the ethanol-treated WT and TLR4-KO mice were similar in both groups, and reached a peak of 125 ± 20 mg/dl.

After 2 months, the control and ethanol-treated females were exposed overnight to males, and the appearance of a copulation plug was considered to be embryonic day 0 (E0). After mating, pregnant mice were individually housed and dams were maintained with solid diet and either water or 10% ethanol solution during gestation and lactation as previously reported [32]. We also used a sucrose pair feeding group during gestation and lactation. However, this group was not included in the study since dams drank the sucrose solution during the first 2 h of the dark cycle, and avoided any liquid for the rest of the day, and food consumption also reduced. Consequently, the body weight gain during gestation did not increase as it did in the control or ethanol-fed groups.

At 15 days of gestation, some dams were anesthetized, killed to obtain embryos (males and females were used), and maternal samples (blood, amniotic fluid, cortex). The other pregnant mice were maintained throughout gestation and lactation. At birth, litters were culled to 6–8 pups per dam, with approximately 45–50% females. Male and female pups were maintained with the dam, although female pups were used in most experiments. At postnatal day (PND) 25, pups were weaned and maintained with tap water and solid food ad libitum.

For the biochemical studies, the cerebral cortices from embryos on E15 (*n* = 8 embryos/group) and pups on PND 0 (*n* = 8 pups/group), 20 (*n* = 8 pups/group), and 66 (*n* = 8 pups/group) were used. For the electron microscopy and immunohistochemistry studies, cortices of pups on PND 20 (*n* = 10 pups/group) were also used. Finally, the behavior studies were conducted in mice on PND 66 (*n* = 11–15 mice/group).

The cortex and maternal serum samples (obtained from the hepatic portal vein) were immediately snap-frozen in liquid nitrogen and stored at −80 °C until further analysis were performed.

All the experimental procedures were approved by the Ethical Committee of Animal Experimentation of the Príncipe Felipe Research Center (Valencia, Spain) and were carried out in accordance with the guidelines approved by the European Communities Council Directive (2010/63/EU) and Spanish Royal Decree 1201/2005.

### Evaluation of maternal care

Maternal care was assessed by evaluating time spent in the nest, maternal grooming (frequency and duration), and care toward their own pups. These experiments were performed at PND 3–5 between 10:00 h and 13:00 h, and were monitored by videotape, as previously described [33].

### ELISA analysis

Protein lysates from the cortices were obtained by homogenization (1% Nonidet P-40, 20 mM Tris–HCl, pH8, 130 mM NaCl, 10 mM NaF, 10 g/ml aprotinin, 10 g/ml leupeptin, 10 mM DTT, 1 mM Na_3_VO_4_, and 1 mM PMSF). Total protein concentrations were determined by the Pierce BCA Protein Assay Kit (ThermoFisher Scientific, Barcelona, Spain), and homogenized extracts were used to determine MAP-2 levels (Biomatik, Wilmington, USA). Serum, amniotic fluid, and cortex samples were also used to analyze the levels of IL-17, MCP-1 (monocyte chemoattractant protein-1) and MIP-1α (macrophage inflammatory proteins-1α) (Peprotech, Barcelona, Spain), IL-1β (eBioscience, Vienna, Austria) and fractalkine (also named CX_3_CL1, R&D Systems, Abingdon, UK) following the manufacturer’s instructions. For the ELISA cytokine determination in the supernatant of the brain homogenate, a spike/recovery assay was performed [34] using known concentrations of the different standards of cytokines or chemokines in the brain homogenate samples.

### Western blot analysis

The western blot technique was performed in cerebral cortex tissue lysates, as previously described [28]. The primary antibodies used were PLP (proteolipid protein), MBP (myelin basic protein), major histocompatibility complex class II (MHC-II), CD11b (Abcam, Cambridge, UK), synaptotagmin, synapsin IIa (BD Bioscience, Madrid, Spain), Tuj-1 (Neuromics, Minneapolis, USA), and caspase 3 (active fragment of 17 kDa) (Cell Signaling, Massachusetts, USA). Membranes were washed, incubated with the corresponding HRP-conjugated secondary antibodies, and developed with the ECL system (ECL Plus; Thermo Fisher Scientific, Illinois, USA). All the membranes were stripped and incubated with GAPDH (glyceraldehyde 3-phosphate dehydrogenase) as a loading control (Chemicon, California, USA). Band intensity was quantified with the ImageJ 1.44p analysis software (National Institutes of Health, USA). The densitometry analysis is shown in arbitrary units normalized to the loading control. Additional file 1: Table S1 shows that the basal values of the various proteins evaluated by western blotting in the pup’s cortices display no significant differences when comparing both the WT and TLR4-KO groups.

### Brain tissue preparation and electron microscopy

Mice (PND 20) were anesthetized by an intraperitoneal injection of sodium pentobarbital (0.06 mg/kg) for analgesia. Then they were perfused transcardially with 0.9% saline containing heparin, followed immediately by 2% paraformaldehyde (PF) and 2.5% glutaraldehyde in 0.1 M phosphate buffer, pH 7.4, for tissue fixation. The PF-fixed brains were removed, post-fixed overnight at 4 °C with the same fixative solution, and then stored at 4 °C in PBS. Coronal 200-μm sections of the cortex (used comparable stereotaxic coordinates on the adult brain, from Bregma 3.08 mm to Bregma 2.58 mm) were cut on a vibratome Leica VT-1000 (Leica, Heidelberg, Germany). Sections were post-fixed with 2% osmium, rinsed, dehydrated, and embedded in Durcupan resin (Fluka, Sigma-Aldrich, St. Louis, USA). Semithin sections (1.5 μm) were cut with an Ultracut UC-6 (Leica, Heidelberg, Germany) and were stained lightly with 1% toluidine blue. Finally, ultra-thin sections (0.08 μm) were cut with a diamond knife, were stained with lead citrate (Reynolds solution), and were examined under a transmission FEI Tecnai G2 Spirit electron microscope (FEI Europe, Eindhoven, Netherlands) using a digital camera Morada (Olympus Soft Image Solutions GmbH, Münster, Germany). Finally, images were analyzed by the MetaMorph software analysis (version 7.0). Quantification was carried out on 4–5 sections per cortex with 5 mice/group. For each group, 150–175 postsynaptic density thicknesses were analyzed, and the numbers of synaptic vesicles were quantified in 75–100 presynaptic terminals.

### Brain tissue preparation and immunohistochemistry

Mice (PND 20) were anesthetized by an intraperitoneal injection of sodium pentobarbital (0.06 mg/kg). Mice were perfused transcardially with 0.9% saline containing heparin, followed immediately by 4% PF in 0.1-M phosphate buffer, pH 7.4, for tissue fixation. Then brains were placed inside histology cassettes and were processed for permanent paraffin embedding in a Leica ASP 300 tissue processor (Leica Microsystems). The processor performed the following steps: 60 min in formalin, 45 min in 70% ethanol, 45 min in 90% ethanol, four changes in 100% ethanol (one for 45 min and three for 60 min, respectively), three changes in xylene (45, 60, and 75 min, respectively), and three changes in paraffin (Histowax, melting point 56-58 °C) for 60 min. Five micrometer-thick paraffin-embedded sections (5 μm) from the cortex (used comparable stereotaxic coordinates on the adult brain, Bregma 3.08 mm to Bregma 2.58 mm) were cut and mounted on coated slide glass. Tissue sections were then processed with the Envision Flex + kit (DAKO) by blocking endogenous peroxidase activity for 5 min and then by incubating with the following primary antibodies: PLP (proteolipid protein), MBP (myelin basic protein) (Abcam, Cambridge, UK), and Iba-1 (Wako Chemicals, Neuss, Germany). The reaction was visualized by Envision Flex + horseradish peroxidase for 20 min and finally by diaminobenzidine for 10 min. Sections were counterstained with Mayer’s hematoxylin (DAKO S3309; ready to use) for 5 min. Immunostaining was visualized and digitally recorded with a Pannoramic 250 digital slide scanner (3DHistech, Budapest, Hungary). Protein immunoreactivity was quantified using the MetaMorph Imaging Series 7.0 analysis software. Quantification was assessed by measuring the thresholded area occupied by the positive staining of these proteins in relation to the whole areas of tissue in the brain sections. Approximately, five fields of each brain section of at least five animals per group were analyzed.

### Behavioral testing

#### Elevated plus maze

An elevated plus maze test was carried out in the dark phase, following the procedure described by [35]. Briefly, this test consisted in two open arms (OAs) (30 × 5 × 0.25 cm) and two closed arms (CAs) (30 × 5 × 15 cm) with a central platform (5 × 5 cm). The entire apparatus was raised 45 cm above floor level. The measurements taken during the test period were frequency of entries, and the time and percentage of the time spent in each section of the apparatus.

#### Passive avoidance test

Step-through inhibitory avoidance apparatus for mice (Ugo Basile, Comerio-Varese, Italy) was employed for the passive avoidance test. This test was also conducted in the dark cycle. The cage is made of Perspex sheets and is divided into two compartments (15 × 9.5 × 16.5 cm each). The safe compartment is white and illuminated by a light fixture (10 W) fastened to the cage lid, whereas the “shock” compartment is dark and made of black Perspex panels. On the day of training, each mouse was placed in the illuminated compartment. After a 60-s period of habituation, the door leading to the dark compartment was opened. When the animal had placed all four paws in the dark compartment, a foot shock (0.5 mA, 3 s) was delivered, and the animal was immediately removed from the apparatus and returned to its home cage. The time taken to enter the dark compartment (step-through latency) was recorded. Retention was tested 24 and 72 h later following the same procedure, but without shock. The maximum step-through latency was 300 s.

### Statistical analysis

The results are reported as mean ± SEM. Statistical analyses were performed using GraphPad Prism v7.01 (GraphPad SoftwareInc., La Jolla, CA, USA) or the IBM Statistical Package for Social Sciences, statistical version 19.0 (IBM, Armonk, NY, USA). Statistical significance for the biochemical studies was determined by a two-way ANOVA with Tukey’s multiple comparisons and a three-way ANOVA to study gender differences between groups. The elevated plus maze test data were analyzed by an ANOVA with two “between” subject variables—“Genetics” with two levels (WT and KO) and “Treatment”—with two levels (ethanol exposure or not). The passive avoidance data were analyzed using a mixed ANOVA with these two “between” variables—“Genetics” and “Treatment”- and a “within” subject variable: “Days” (training, a 24-h test and a 72-h test). Bonferroni adjustment was employed to make post hoc comparisons.

## Results

### Animal model, pregnancy characteristics, maternal care, and offspring body and brain weights

For the animal model, female mice were given alcohol before and during gestation and lactation since women who consume alcohol during gestation are usually drinkers before gestation [36], and many of them are also drinkers during lactation [37–39].

Table 1 shows the different parameters of the four groups of pregnant dams used: WT or TLR4-KO, with or without ethanol treatment. As shown in this table, the daily food and liquid intakes were similar for the ethanol-treated or for the untreated WT and TLR4-KO mice. Although ethanol drinking represented approximately 10–12% of the total calorie intake in pregnant dams, the body weight gain during the gestation period was similar in the WT and TLR4-KO mice, both with and without ethanol treatment. Furthermore, no signs of dehydration were observed in ethanol drinking mice (see “Methods” section). No significant differences were noted in the number of pups born of the control and ethanol-drinking WT or TLR4-KO mice dams (Table 1). Nevertheless, the body weight of newborn WT pups exposed prenatally to ethanol showed significant reduction compared with the controls pups. Notably, similar body weights were noted in newborn TLR4-KO pups exposed or not to ethanol during gestation.

**Table 1:** Characteristics of mice during the gestational period

	Control WT	Ethanol WT	Control TLR4-KO	Ethanol TLR4-KO
Maternal body weight (g)^a^	35.45 ± 0.40	34.26 ± 0.27	34.80 ± 0.26	34.55 ± 0.81
Gestational weight gain	11.68 ± 1.71	11.37 ± 1.25	12.03 ± 1.24	11.38 ± 1.10
Body weight at PND 0 (g)	1.38 ± 0.04	1.32 ± 0.06*	1.39 ± 0.08^#^	1.37 ± 0.01^#^
Pups number at birth	7.63 ± 1.41	6.00 ± 2.00	7.00 ± 2.00	7.00 ± 1.73
Total food intake (g)^b^	3.97 ± 0.33	3.86 ± 0.12	4.05 ± 0.06	4.02 ± 0.19
Total liquid intake (mL)^b^	4.16 ± 0.39	4.32 ± 0.15	3.80 ± 0.21	3.77 ± 0.16
Total ethanol intake (g)^b^	–	0.34 ± 0.01	–	0.30 ± 0.15

*p < 0.05 compared to the control WT group

#*p* < 0.05 compared to the ethanol-treated WT group

^a^Maternal body weight at the end of the gestational period

^b^Total food, liquid, and ethanol intake per mice and day

We also assessed maternal behavior by evaluating maternal care by monitoring the time spent in the nest and the grooming time. The ANOVA for the maternal behaviors reveals no differences between the groups in the percentage of time spent in the nest and the percent time grooming and care toward pup (Table 2). These data suggest that the reduction in body weight noted in the ethanol-exposed pups was not due to deficient maternal care of the alcohol-drinking (WT, TLR4-KO) dams.

**Table 2:** Maternal care behavior

	WT	WT PPEE	TLR4-KO	TLR4-KO PPEE
Percent time on nest (min/2 h observation)	45 ± 5	41 ± 4	49 ± 3	51 ± 2
Percent time grooming and care toward pup	24 ± 6	19 ± 1	22 ± 5	20 ± 3

### Maternal immune activation and the cytokine/chemokine profile during pregnancy and in the cerebral cortices of fetuses and offspring: role of the TLR4 response

**Fig. 1:**
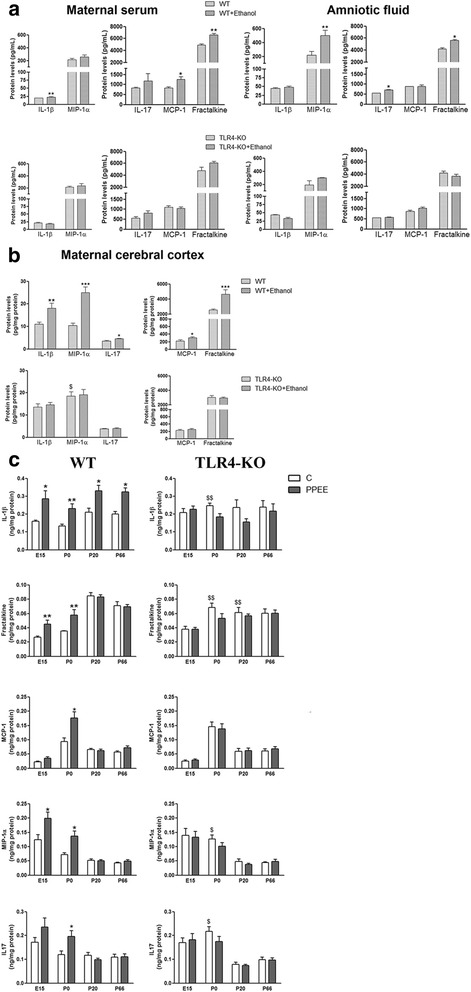
Role of TLR4 in the expression of cytokines (IL-1β, IL-17) and chemokines (fractalkine, MCP-1, MIP-1α) in (**a**) maternal serum and amniotic fluid on E15, (**b**) in the maternal cerebral cortices of the WT and TLR4-KO pregnant female dams which underwent, or did not, chronic ethanol consumption, and (**c**) in the cerebral cortices of the WT and TLR4-KO embryos and pups on E15 and PND 0, 20, and 66 exposed, or not, to ethanol during the embryonic and postnatal periods. *PPEE* prenatal and postnatal ethanol exposure. Data represent mean ± SEM, *n* = 8 mice/group. *p < 0.05, **p < 0.01, ***p < 0.001, compared to their respective control group, $*p* < 0.05, $$*p* < 0.01 compared to their corresponding control WT group

We next evaluated the levels of the cytokines/chemokines in maternal serum and amniotic fluid on E15. Figure 1a shows that whereas the levels of IL-1β, MCP-1 and fractalkine significantly increased in maternal serum, MIP-1α, IL-17, and fractalkine were upregulated in both the amniotic fluid and cortices of the WT 15-day-old fetuses exposed to ethanol (IL-1β, MIP-1α and fractalkine) (Fig. 1c). Notably, the same cytokines along with MCP-1 and IL-17 were significantly upregulated in maternal cortices (Fig. 1b) and in cortices of newborn (day 0, IL-1β, fractalkine, MIP-1α, and IL-17) and PND 20 female pups (Fig. 1c). It is interesting to note that only the pro-inflammatory cytokine IL-1β was maintained for the long term in the cortices of the WT mice on PND 20 and 66 (Fig. 1c).

The results in Fig. 1a, b, c revealed that elimination of TLR4 abolished the induction of cytokines/chemokines in maternal serum, amniotic fluid and the cerebral cortex (Fig. 1a–b), and in the cortices of both fetuses and offspring at PND 20 and PND 66 (Fig. 1c). It is important to note that the basal levels of some cytokines/chemokines differed between WT and TLR4-KO. For instance, the two-way ANOVA analyses revealed that the levels of some cytokines in the cortices of pups at PND 0, PND 20, and the maternal cerebral cortex differed between both groups (see Additional file 2: Table S2).

Because we have previously observed sex differences in ethanol-induced neuroinflammation [29, 40], we also assessed the cortical cytokine/chemokine levels in male pups (PND 0 and PND 20). Additional file 1: Figure S1 shows that prenatal and postnatal exposure to ethanol increased the levels of fractalkine in the pups of PND 0, and MCP-1 and MIP-1α in the pups of PND 20. The three-way ANOVA analyses (Additional file 2: Table S3) revealed that gender had a significant effect for MIP-1α, MCP-1, and fractalkine in the pups at PND 0 and 20, for IL-17 at PND 0. A significant effect of gender and genotype was also found for MCP-1 in the pups at PND 20 (Additional file 1: Figure S1). These gender differences were associated with increased neuroinflammation in female pups (PND 0 and 20) compared to males, which were prenatally and postnatally exposed to ethanol.

### The TLR4 response participates in ethanol-induced structural and cellular dysfunctions in the developing brain

**Fig. 2:**
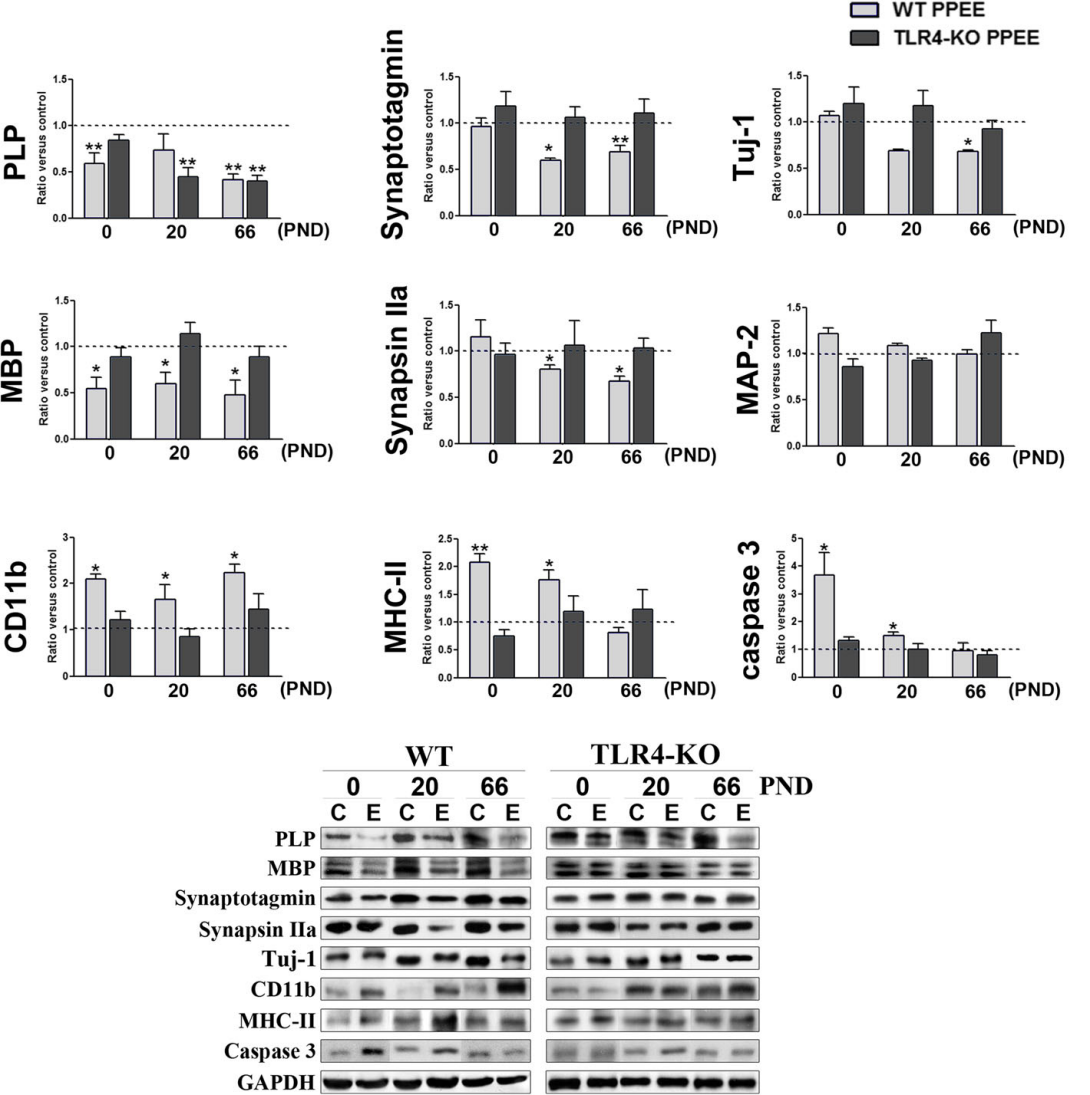
Role of TLR4 in the expression of several proteins induced by prenatal and postnatal ethanol exposure. Data represent the immunoblot analysis of PLP, MBP, synaptotagmin, synapsin IIa, Tuj-1, CD11b, MHC-II, caspase 3 (active fragment of 17 kDa), and the ELISA analysis of MAP-2 from the cerebral cortices of the WT and TLR4-KO pups on PND 0, 20, and 66 exposed (E), or not (C), to ethanol during the embryonic and postnatal periods. A representative immunoblot of each protein is shown. *PPEE* prenatal and postnatal ethanol exposure. Data represent mean ± SEM, *n* = 8 mice/group. *p < 0.05, **p < 0.01, ***p < 0.001, compared to their respective control group

To evaluate the association between the activation of the TLR4 innate immune response with structural and cellular changes induced by ethanol exposure, we assessed the levels of several protein markers of glial and neuronal cells, along with myelin and synaptic proteins. The experiments were performed in the cortices of the WT and TLR4-KO pups on PND 0, 20, and 66, either exposed or not prenatally and postnatally to ethanol. Figure 1c shows that the upregulation of cytokines/chemokines was associated with microglia activation, as demonstrated by the increase in the markers: CD11b in the cerebral cortices of the WT offspring on PND 0, 20 and 66, and MHC-II in the WT pups on PND 0 and 20 (Fig. 2). We also observed that ethanol treatment significantly downregulated myelin-related proteins, such as PLP and MBP, in the WT pups on PND 0, 20, and 66 (Fig. 2), and in the levels of the immature neuronal marker Tuj-1 in the cerebral cortices of the ethanol-exposed WT pups on PND 20 and 66, although we found no changes in MAP-2, a neuronal marker. Likewise, we also assessed the levels of some synaptic proteins, such as synapsin IIa, syntaxin 4, SNAP-25, and synaptotagmin. However, significant differences were noted only in the expression of synaptotagmin and the synapsin IIa levels, at which a significant reduction took place in the cortices of the WT pups on PND 0, 20, and 66 (Fig. 2).

The two-way ANOVA analysis revealed that ethanol exposure during prenatal and postnatal periods did not significantly affect the levels of proteins associated with myelin integrity (MBP), glial activation, and synaptic proteins in the cortices of the TLR4-KO pups exposed to alcohol compared with the untreated control pups. However, a drop in the myelin protein PLP levels was noted in the cerebral cortices of the TLR4-KO pups (PND 20 and 66) exposed to ethanol (Fig. 2) (see Additional file 2: Table S4).

**Fig. 3:**
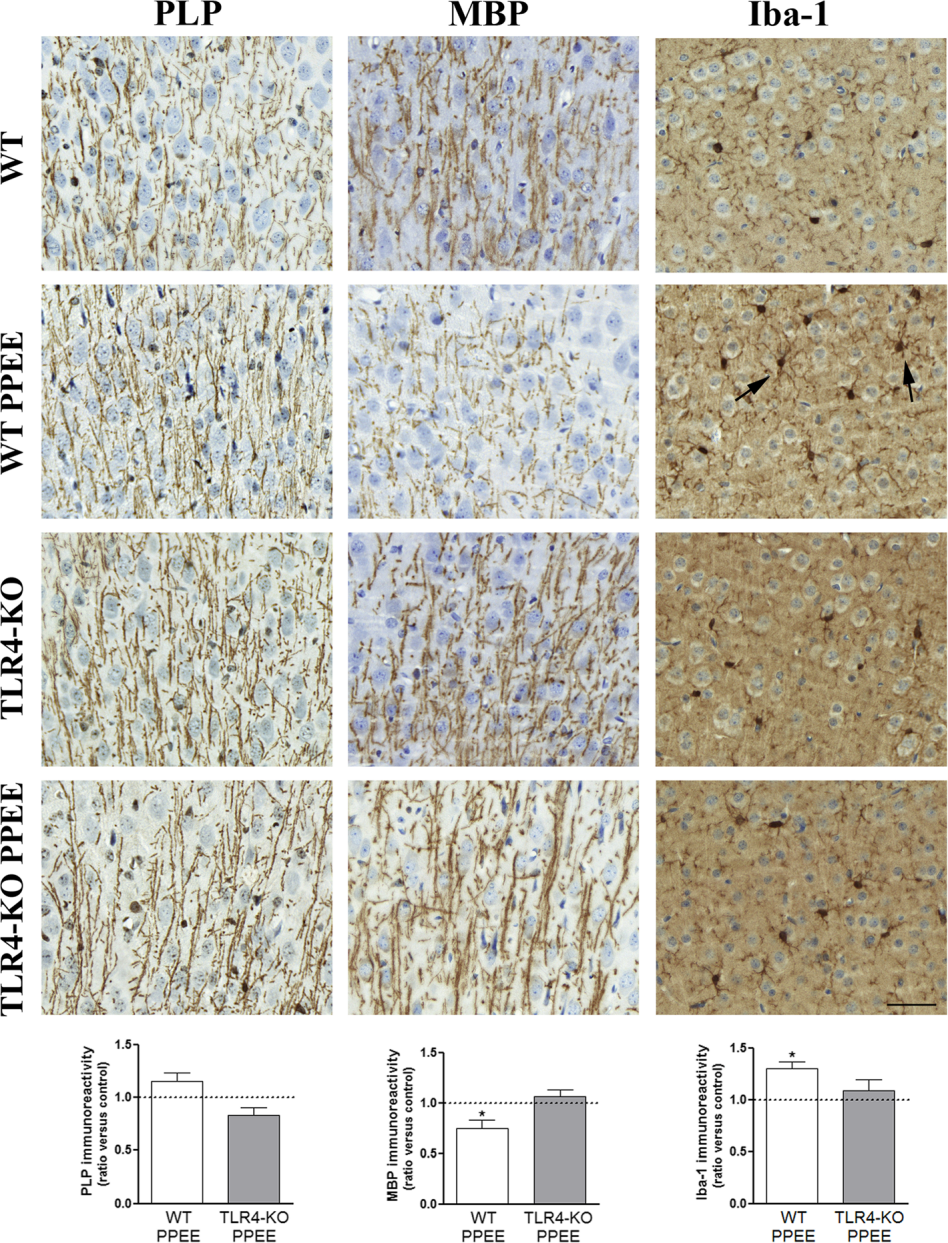
Role of TLR4 in prenatal and postnatal ethanol exposure in PLP, MBP, and Iba-1 immunoreactivity in the cortices of the WT and TLR4-KO pups. *Arrows* show examples of microglial activation. The scale bar is 50 μm. *Bars* represent the quantification values of PLP, MBP, and Iba-1 immunoreactivity, expressed as the thresholded area occupied by specific staining in the cortices of the WT and TLR4-KO mice treated, or not, prenatally and postnatally with ethanol (PPEE). Results are given as means ± SEM (*n* = 5). *p* < 0.05 compared to the ethanol-treated WT or TLR4-KO mice with their respective untreated control groups

To confirm that ethanol exposure affects myelin proteins and triggers microglia activation, we also performed an immunohistochemistry analysis in the cortices of the control (WT, TLR4-KO) and ethanol-exposed pups at PND 20. Figure 3 shows the levels and distribution of myelin proteins (PLP, MBP), and microglia activation (Iba-1). The two-way ANOVA analysis revealed that prenatal and postnatal ethanol exposure significantly decreased MBP immunoreactivity, but increased Iba-1 immunoreactivity in the cortices of the WT pups at PND 20. However, no significant changes in the immunoreactivity of these proteins were observed in the control and ethanol-exposed TLR4-KO pups (see Additional file 2: Table S4).

We also measured the 17 kDa active caspase-3-cleveage peptide in the WT and TLR4-KO pups prenatally and postnatally exposed to ethanol. Figure 2 shows that while pre- and postnatal ethanol exposure significantly increased active caspase-3 levels (17 kDa) in the cortices of the WT pups on PND 0 and 20, no significant changes in active caspase-3 levels were observed in the TLR4-KO pups exposed to ethanol (see Additional file 2: Table S4).

**Fig. 4:**
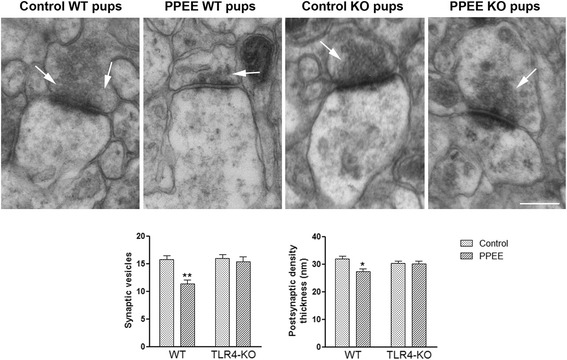
The electron microscopy analysis shows the role of TLR4 in the synaptic structure alteration in the cerebral cortices of the pups (PND 20) exposed to ethanol. The representative transmission electron micrographs from the cortices of the WT and TLR4-KO pups on PND 20 exposed, or not, to ethanol during the prenatal and postnatal periods are shown. *Arrows* mark vesicles. *PPEE* prenatal and postnatal ethanol exposure. *Scale bar* represents 250 nm. *Bars* represent the vesicle number and postsynaptic density thickness. Data represent mean ± SEM, *n* = 5 pups/group. *p < 0.05, compared to their respective control group

The electron microscopy studies (Fig. 4) further showed that pre- and postnatal ethanol exposure significantly reduced the vesicle number and postsynaptic density thickness in the WT pups on PND 20. No significant changes were observed between the TLR4-KO pups (PND 20) exposed to ethanol (Fig. 4) (see the two-way ANOVA analysis in Additional file 2: Table S4).

### Prenatal ethanol exposure causes long-term behavioral dysfunctions associated with the TLR4 response

To assess whether ethanol exposure during brain development is capable of inducing long-term behavioral impairments in adult mice, we used elevated plus maze and passive avoidance tests.

#### Elevated plus maze

The elevated plus maze data are presented in Table 3. The ANOVA showed an effect on the time [F(1,46) = 4.286; *p* < 0.05] and the percentage of time [F(1,46) = 23.640; *p* < 0.001] spent in the OAs of the maze for the genetics × treatment interaction. The WT mice exposed to ethanol during the prenatal and postnatal periods spent less time (*p* < 0.01) and a lower percentage of time (*p* < 0.001) in the OAs than both their control counterparts and the ethanol-exposed TLR4-KO mice (*p* < 0.01 and *p* < 0.001, respectively). Furthermore, the ANOVA for the time spent in the CAs revealed an effect for the variables treatment [F(1,46) = 8.289; *p* < 0.01] and genetics [F(1,46) = 5.891; *p* < 0.05], and for the genetics × treatment interaction [F(1,46) = 4.926; *p* < 0.05]. The WT mice exposed to ethanol spent more time in the CAs than their respective control group (*p* = 0.001) and the TLR4-KO mice exposed to ethanol (*p* < 0.01). This finding suggests that ethanol exposure induces anxiety-related behavior.

**Table 3:** Role of TLR4 in the EPM in WT and TLR4-KO mice at PND 66 exposed or not to ethanol during the gestational and postnatal period

	WT	WT PPEE	TLR4-KO	TLR4-KO PPEE
Time in OA (s)	98 ± 9	24 ± 6**	101 ± 13	101 ± 30^++^
% time in OA	43 ± 4	10 ± 3***	44 ± 14	40 ± 10^+++^
Time in center (s)	72 ± 7	57 ± 9	69 ± 8	61 ± 16
Time in CA (s)	130 ± 1	220 ± 13***	126 ± 14	138 ± 26^++^
OA entries	10 ± 2	2 ± 1	8 ± 1	2 ± 1
% OA entries	46 ± 3	21 ± 3	56 ± 3	50 ± 9
Closed entries	12 ± 1	8 ± 1	6 ± 1	4 ± 1
Total entries	22 ± 2	10 ± 1	14 ± 1	6 ± 1

*OA* open arms and *CA* closed arms. Data are presented as mean (± SEM), *n* = 11–15 mice/group. **p < 0.01, ***p < 0.001, as compared to the control WT mice; ++*p* < 0.01, +++*p* < 0.001, as compared to the ethanol WT mice. *PPEE* prenatal and postnatal ethanol exposure

#### Passive avoidance test

**Fig. 5:**
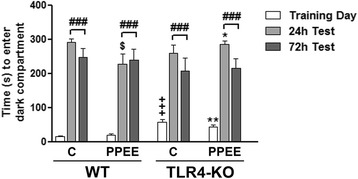
Role of TLR4 in the passive avoidance test in the WT and TLR4-KO mice on PND 66 exposed, or not, to ethanol during the gestational and postnatal periods. *Bars* represent the time taken to enter the dark compartment during the passive avoidance test on the training day and during the test sessions (24 and 72 h after training). *PPEE* prenatal and postnatal ethanol exposure. Data are presented as mean (±SEM), *n* = 15 mice/group. +++*p* < 0.001, compared with the control WT mice during the training session; ###*p* < 0.001, compared with the respective group on the training day; *p > 0.05, **p < 0.01, compared with the ethanol pre-treated WT mice during the training session or the 24-h test; $*p* < 0.05, compared with their corresponding control pretreated group of mice on the 24-h test day

The memory function evaluated with the passive avoidance test was based on the association formed between an aversive stimulus, such as mild foot shock, and a specific environmental context. This test measures short- and long-term memory. The ANOVA revealed an effect on the variable days [F(2,88) = 205.254; *p* < 0.001] as animals in the test sessions at 24 and 72 h took longer latency to cross to the dark compartment than on the training day (*p* < 0.001, in all cases) (Fig. 5). The statistical analysis also showed an effect of the genetics × treatment × days interaction [F(2,88) = 2.997; *p* = 0.05]. On the training day, the TLR4-KO saline- or ethanol-treated mice showed longer latencies to enter the dark compartment than their corresponding WT (*p* < 0.001 and *p* < 0.01, respectively). The WT ethanol-treated mice showed shorter latencies in the test at 24 h than their respective control group (*p* < 0.05) and the TLR4-KO prenatally ethanol-exposed mice (*p* < 0.05), suggesting that ethanol exposure induces learning and memory dysfunctions.

## Discussion

Ethanol is considered one of the commonest substances that impact the developing brain [41]. Indeed a large body of evidence indicates that alcohol exposure during brain ontogeny causes long-term structural and neurobehavioral consequences in humans and experimental animals (see refs. [4, 42, 43]). Although the actions of ethanol during brain ontogeny are complex and different mechanisms have been proposed [5, 44], inflammation is now recognized as being a critical contributor to both normal development and injury outcome in the immature brain [19], and recent studies indicate the role of neuroinflammation in alcohol-induced adult and adolescent brain damage [45]. The present findings provide evidence that alcohol consumption during pregnancy triggers a maternal and fetal immune response by inducing microglial activation along with the production of cytokines/chemokines in fetal and postnatal brains. These effects might be associated with structural alterations in the cortical myelin and synaptic proteins, as well as with long-term behavior dysfunctions. We also demonstrate the critical role of the TLR4 response since mice deficient in the TLR4 function were protected against ethanol effects during brain development.

Studies in human and experimental animals have reported the vulnerability of the developing brain to toxic effects of ethanol by demonstrating that alcohol abuse during pregnancy can cause permanent brain damage in offspring, which is associated with life-long behavioral, social, and cognitive disorders (e.g., [4, 43, 46]). Despite the different mechanisms proposed to explain the pathology of FASD, growing evidence indicates that maternal immune activation can affect the CNS structure and function in offspring and cause both acute and long-lasting changes in behavior [47]. The present study shows that maternal alcohol drinking not only triggers the release of cytokines/chemokines in maternal blood (IL-1β, MCP-1, and fractalkine) and the brain but also upregulates the levels of MIP-1α, IL-17, and fractalkine in amniotic fluid, and in the cortices of the WT 15-day-old fetuses exposed to ethanol (IL-1β, MIP-1α, and fractalkine). It is important to reinforce that IL-1β and IL-17 are important pro-inflammatory cytokines and their overexpression is associated with brain injury. For instance, IL-1β has been associated with systemic inflammation that contributes to acute brain injury [48], while IL-17 blocks neural stem cell proliferation and reduces the number of astrocytes and oligodendrocyte precursor cells by playing a direct role in blocking remyelination and neural repair in CNS damage [49]. Alterations in the mother’s immune system and elevated maternal IL-17 levels have also been seen to promote abnormal cortical development and to cause behavioral abnormalities in offspring [50].

According with our experimental results, human studies have also shown that alcohol abuse during human pregnancy induces upregulation of cytokines (IL-1β, IL-6, and TNF-α) in both maternal blood and fetal cord levels at delivery [51]. These studies support the view that the fetuses from alcohol-drinking mothers are exposed to similar cytokines and chemokines as maternal blood and suggest that presence of cytokines/chemokines in fetal tissue could result from either maternal blood or fetal and/or placental tissues. Experimental evidence indicates that not all cytokines can be transferred to the fetus, while some cytokines, such as IL-6, can be bidirectionally transferred, and TNF-α and IL-1β are minimally transferred to fetal circulation [52]. Placental cells can also produce cytokines and chemokines during infection or under pathological conditions [17, 53]. A recent study has demonstrated that administration of low doses of alcohol to pregnant rat dams from E10 to E16 increases the gene expression of cytokines and chemokines in both the placenta and fetal brain, while the levels of these cytokines are not significantly affected in maternal peripheral blood [17]. Although these results suggest that ethanol can directly impact the fetal and placental immune system response, it is presently unknown whether maternal cytokines could also contribute to the fetal neuroimmune response.

It is noteworthy that the innate immune system and TLRs play important roles during brain development by participating in essential processes [54–56] such as synaptogenesis, neuronal migration, activity-dependent refinement of circuits, or synaptic plasticity. However, the over-activation of this system and glial TLRs [56] by prenatal infection or prenatal immune activation induces changes in the brain function and long-term behavior alterations [47, 57]. In the present study, we provide evidence that prenatal and postnatal alcohol exposure over-activates the innate immune system, in particular the TLR4 response, by triggering microglial activation along with the production of cytokines and chemokines in fetuses and offspring brains. Astrocytes are also active players in neuroinflammation [58], and TLR4 expression upregulation occurs under inflammatory conditions [56]. Previous studies have shown the capability of ethanol to activate the TLR4 signaling response in both astrocytes [59] and microglia [28], which leads to the release of cytokines and inflammatory mediators to create a neuroinflammatory environment that could induce neural cell damage. Here, we show that activation of the neuroimmune system is accompanied with significant alterations in the expression of the brain structural and functional proteins, such as myelin and synaptic-associated proteins as well as with apoptotic proteins, as caspase-3 in cerebral cortex of pups exposed to ethanol. Activation of the neuroimmune system and changes in the cortical structure also correlated with long-term cognitive and behavioral dysfunction in adult mice exposed to ethanol during development, as demonstrated by the deficits in the passive avoidance test and elevated plus maze tests. In agreement with our experimental results, neuroimaging studies and diffusion tensor imaging in FASD children have revealed major alterations in the cortical white matter microstructure, as well as functional connectivity between cortical and deep gray matter structures, and its relation with behavior [43, 46].

In the last few years, experimental studies have also confirmed the participation of the neuroimmune system in FASD [14–16]. However, these preclinical studies focused mainly on the effects of ethanol exposure on the “brain growth spurt” stage, characterized by glial proliferation, dendritic arborization, and cerebellum development. This period, which takes place in rodents during the neonatal period, is the equivalent to the third trimester of human pregnancy [44, 60]. These studies have demonstrated that administration of high doses of ethanol (4 g/kg/day) during PND 4–9 in mice [14] upregulates microglial cells and the production of cytokines/chemokines in the hippocampus, cerebellum, and cerebral cortex, and causes neural damage. Similarly, rat pups, which were administered ethanol (5 g/kg) by intragastric intubation on PND 7–9, induced cytokine production (TNF-α, IL-1β, and TGF-β), increased caspase-3 levels in both the cerebral cortex and hippocampus, and impaired memory performance [15], while ethanol administration during PND 3–5 induced proinflammatory cytokines in both the rat hippocampus and cerebellar vermis, with neuronal loss found only in the hippocampus [18]. Interestingly, blocking microglial activation by administering pioglitazone, an agonist of PPAR (peroxisome proliferator-activated receptor)-γ [14], or resveratrol [15], with anti-inflammatory and antioxidant effects, abrogates the upregulation of brain inflammatory signaling and neuronal death, and prevents some cognitive deficits in neonates. Prenatal exposure to ethanol also leads to the upregulation of some cytokines in the prefrontal cortex and hippocampus of postnatal rats on day 8 [16]. These results support the notion that activation of the neuroimmune system could participate in some cognitive and behavioral deficits associated with FASD.

An important finding of this study is the demonstration that TLR4 response participates in the effects of ethanol during the developing brain, since mice lacking the TLR4 function (TLR4-KO) are protected against ethanol-induced microglia activation, cytokine/chemokine release, myelin and synaptic alterations, and also against the long-term memory and anxiety-like behavior impairments. We noted that despite TLR4-KO mice showing comparable behavioral responses as the WT mice, prenatal and postnatal ethanol treatment induced neither anxiety nor memory dysfunctions in these mice. Conversely, activation of TLR4 by LPS administration to mice on PND 14 increased the expression of the cytokines in both plasma and the brain, and caused long-term anxiety-like behavior [61]. TLR4 is expressed in embryonic, fetal, and postnatal stages, and can be activated by different stimuli, including endogenous compounds (DAMPs) or alcohol [28], by inducing microglial activation, which triggers cytokine production in neonatal rats following hypoxia [62]. It is important to highlight that ethanol or DAMPs activate the same TLR4 signaling pathways as LPS [28], the natural TLR4-ligand, which leads to the activation of transcription factors, NF-κB and AP-1, and interferon pathways [63]. Some DAMPs are released from tissue damage or in response to inflammatory stimuli, such as heat shock proteins (e.g., HSP70 [64]) or the high-mobility group box-1 protein (HMGB1) [65]. Ethanol or endogenous molecules could initiate or amplify the TLR4 response and could lead to neuroinflammation and irreversible neural damage, which can cause long-term behavioral dysfunction. For instance, HMGB1 is upregulated in the brains of ethanol-exposed adolescent animals, and remains elevated from adolescence to young adulthood [10, 66].

We are aware of the limitations of the present study. For instance, using knockout mice during development, neurochemical compensatory changes can influence behavior. However, no detrimental changes in anxiety and aversive memories [67], and spatial reference memory acquisition [68], have been observed in the TLR4-deficient mice. Another limitation is the use of pups exposed to alcohol during gestation and lactation. Future studies will be required to clarify the actions of ethanol on the immune response in different stages of the developing brain. Although we mainly performed experiments with female pups, further studies are required to evaluate the long-term cognitive and behavioral effects in adult males exposed to ethanol during gestation and lactation, and to assess possible gender differences, as we have demonstrated in adolescence [29]. Sex differences have also been observed in fetuses exposed to low alcohol levels, from E10 to 16, in which higher inflammatory gene expression levels were observed in female brains compared to their male counterparts [17], which suggests sex differences in the inflammatory response during fetal development. Sexually dimorphic effects on neuroendocrine and immune functions have also been reported in offspring exposed in utero to alcohol [69, 70].

Finally, ethanol-induced activation and/or dysfunctions in the innate immune/TLR4 response during embryonic/fetal development might have further consequences for immune-related developing organs (e.g., endocrine, liver, gastrointestinal tract and intestinal lymphoid tissues, etc.) by contributing to not only brain dysfunctions but also to other long-lasting pathologies in FASD, such as liver damage [71] and a higher incidence of infections [72, 73], which have been reported in FASD children.

## Conclusions

In summary, the results of the present study highlight the critical role of the TLR4 activation in the release of the cortical cytokines and chemokines induced by ethanol exposure during prenatal and postnatal periods, effects which could be associated with brain development impairment and long-term behavior dysfunctions. Furthermore, the present findings highlight a novel role of the neuroimmune function in prenatal ethanol effects and may offer new therapeutic approaches to attenuate the numerous birth defects associated with prenatal alcohol exposure.

## Additional files

Additional file 1: **Figure S1.** Role of TLR4 in the expression of cytokines (IL-1β, IL-17) and chemokines (fractalkine, MCP-1, MIP-1α) in the cerebral cortices of the WT and TLR4-KO male pups on PND 0 and 20 exposed, or not, to ethanol during the embryonic and postnatal periods. PPEE: prenatal and postnatal ethanol exposure. Data represent mean ± SEM, n = 4 mice/group. *p < 0.05, compared to their respective control group. **Table S1.** Basal level of the brain proteins analyzed by western blotting between untreated or control (C) WT and TLR4-KO pups of PND 0 and PND 66. (ZIP 429 kb)

Additional file 2: **Table S2. **Summary table of the two-way ANOVA of biochemical data (only statistically significant data is shown). **Table S3.** Summary table of the three-way ANOVA to study gender differences in ethanol-treated and non-treated WT and TLR4-KO pups at PND 0 and 20 (only statistically significant data is shown).** Table S4.** Summary table of the two-way ANOVA of western blot, immunohistochemistry, and electron microscopy data (only statistically significant data is shown). (DOC 74 kb)

## Abbreviations

CA: Closed arms
DAMP: Damaged-associated molecular patterns
E: Embryonic day
FASD: Fetal alcohol spectrum disorders
IL: Interleukin
KO: Knockout
MBP: Myelin basic protein
MHC-II: Major histocompatibility complex class II
NF-κB: Nuclear factor-κB
OA: Open arms
PAMP: Pattern-associated molecular patterns
PLP: Proteolipid protein
PND: Postnatal day
PPAR: Peroxisome proliferator-activated receptor
TLR: Toll-like receptor
WT: Wild-type
