# Supplementary figures and images for: TLR4 response mediates ethanol-induced neurodevelopment alterations in a model of fetal alcohol spectrum disorders
Source: J Neuroinflammation. 2017 Jul 24;14:145. doi: 10.1186/s12974-017-0918-2 (PMC5525270; doi:10.1186/s12974-017-0918-2)

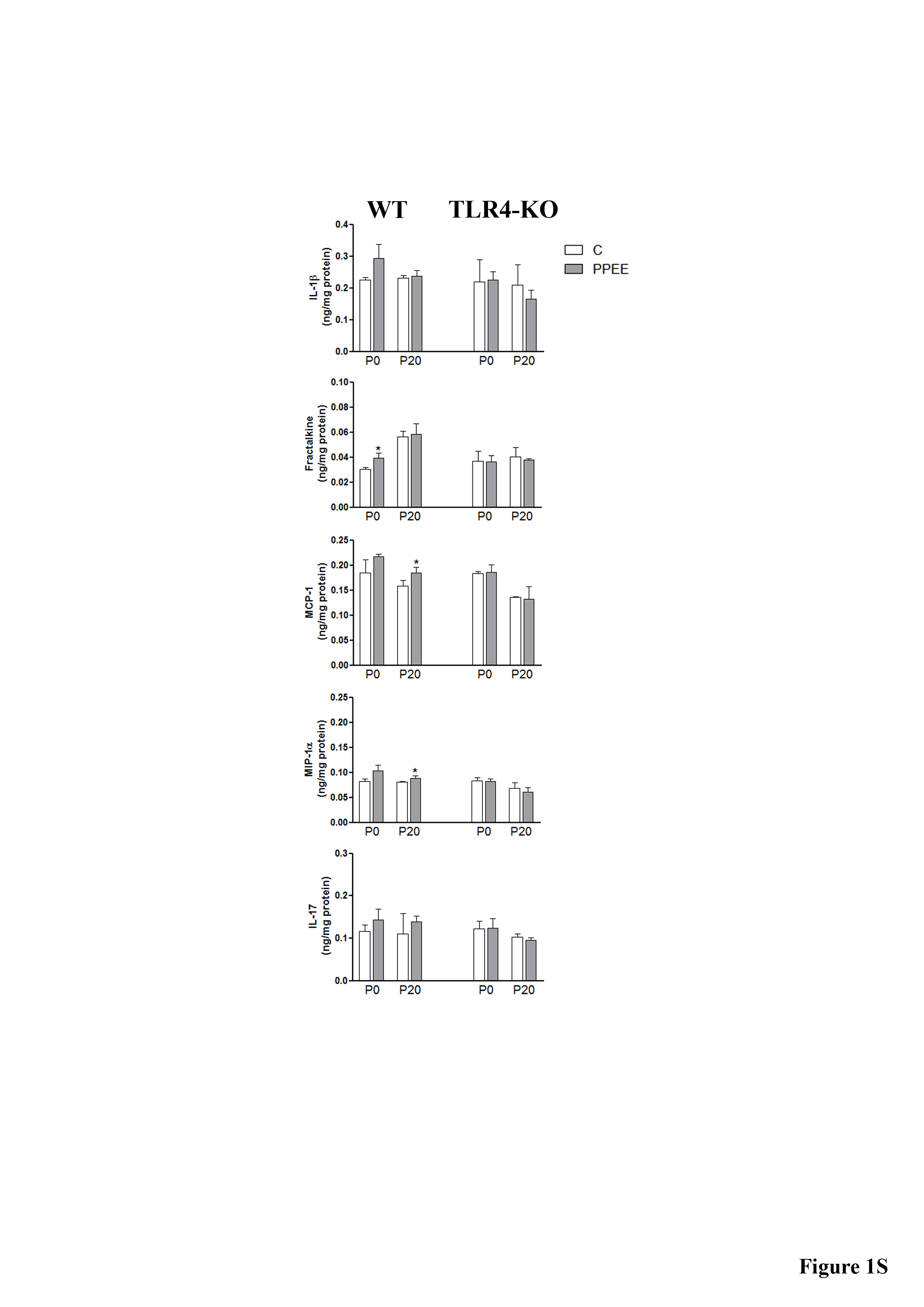

Supplement: Supplementary file 1 — Figure S1. Role of TLR4 in the expression of cytokines (IL-1β, IL-17) and chemokines (fractalkine, MCP-1, MIP-1α) in the cerebral cortices of the WT and TLR4-KO male pups on PND 0 and 20 exposed, or not, to ethanol during the embryonic and postnatal periods. PPEE: prenatal and postnatal ethanol exposure. Data represent mean ± SEM, n = 4 mice/group. *p < 0.05, compared to their respective control group. Table S1. Basal level of the brain proteins analyzed by western blotting between untreated or control (C) WT and TLR4-KO pups of PND 0 and PND 66. (ZIP 429 kb) [file 12974_2017_918_MOESM1_ESM.zip › Fig 1S.tif]
