# Supplementary material for: TLR4 response mediates ethanol-induced neurodevelopment alterations in a model of fetal alcohol spectrum disorders
Source: J Neuroinflammation. 2017 Jul 24;14:145. doi: 10.1186/s12974-017-0918-2 (PMC5525270; doi:10.1186/s12974-017-0918-2)
Supplement: Supplementary file 2 — Table S2. Summary table of the two-way ANOVA of biochemical data (only statistically significant data is shown). Table S3. Summary table of the three-way ANOVA to study gender differences in ethanol-treated and non-treated WT and TLR4-KO pups at PND 0 and 20 (only statistically significant data is shown). Table S4. Summary table of the two-way ANOVA of western blot, immunohistochemistry, and electron microscopy data (only statistically significant data is shown). (DOC 74 kb) [file 12974_2017_918_MOESM2_ESM.doc]

**Additional file 2**

**Table S2.** Summary table of the two-way ANOVA of biochemical data (only statistically significant data is shown).

| **Maternal serum** | Treatment | | Genotype | Interaction | |
| --- | --- | --- | --- | --- | --- |
| IL-1β  MCP-1  Fractalkine | F(1,28) = 4.391; p < 0.05  F(1,28) = 20.281; p < 0.001 | F(1,28) = 7.768; p < 0.01 | | | F(1,28) = 13.350; p < 0.01  F(1,28) = 6.996; p < 0.05 |

| **Amniotic fluid** | Treatment | | Genotype | Interaction | |
| --- | --- | --- | --- | --- | --- |
| IL-17  MIP-1α  Fractalkine | F(1,28) = 7.903; p < 0.01  F(1,28) = 9.355; p < 0.01  F(1,28) = 7.995; p < 0.01 | F(1,28) = 6.975; p < 0.01  F(1,28) = 11.130; p < 0.01 | | | F(1,28) = 7.289; p < 0.05 |

| **Maternal cerebral cortex** | Treatment | | Genotype | Interaction | |
| --- | --- | --- | --- | --- | --- |
| IL-1β  MCP-1  Fractalkine  IL-17  MIP-1α | F(1,28) = 7.671; p < 0.01  F(1,28) = 6.236; p < 0.05  F(1,28) = 9.142; p < 0.01  F(1,28) = 5.842; p < 0.05  F(1,28) = 13.591; p < 0.001 |  | | | F(1,28) = 4.364; p < 0.05  F(1,28) = 10.720; p < 0.01  F(1,28) = 11.534; p < 0.01 |
| **E15 cortex** | Treatment | Genotype | | | Interaction |
| IL-1β  MCP-1  Fractalkine  MIP-1α | F(1,28) = 7.691; p < 0.01  F(1,28) = 4.636; p < 0.05  F(1,28) = 6.371; p < 0.05  [F(1,28) = 4.796; p < 0.05 |  | | | F(1,28) = 6.720; p < 0.05  F(1,28) = 6.165; p < 0.05  F(1,28) = 6.395; p < 0.05 |
| **P0 cortex** |  |  | | |  |
| IL-1β  Fractalkine  IL-17  MIP-1α |  |  | | | F(1,28) = 19.250; p < 0.001  F(1,28) = 14.170; p < 0.001  F(1,28) = 6.992; p < 0.05  F(1,28) = 10.35; p < 0.01 |
| **P20 cortex** |  |  | | |  |
| IL-1β  Fractalkine |  | F(1,28) = 5.827; p < 0.05  F(1,28) = 26.701; p < 0.001 | | | F(1,28) = 10.654; p < 0.05 |
| **P66 cortex** |  |  | | |  |
| IL-1β |  |  | | | F(1,28) = 5.573; p < 0.05 |

**Table S3.** Summary table of the three-way ANOVA to study gender differences in ethanol-treated and non-treated WT and TLR4-KO pups at PND 0 and 20 (only statistically significant data is shown).

| **0 days** | Gender | | Treatment | | Genotype x treatment | Gender x genotype x treatment |
| --- | --- | --- | --- | --- | --- | --- |
| IL-1β  Fractalkine  MCP-1  MIP-1α  IL-17 | F(1,24) = 20.790; p < 0.001  F(1,24) = 21.200; p < 0.001  F(1,24) = 7.416; p < 0.05  F(1,24) = 12.15; p < 0.01 | F(1,24) = 5.568; p < 0.05 | | F(1,26) = 5.806; p < 0.05  F(1,24) = 8.996; p < 0.01  F(1,24) = 6.608; p < 0.05  F(1,24) = 12.46; p < 0.01  F(1,24) = 6.127; p < 0.05 | | F(1,24) = 4.667; p < 0.05 |

| **20 days** | Gender | | Genotype | Genotype x treatment | | Gender x genotype |
| --- | --- | --- | --- | --- | --- | --- |
| IL-1β  Fractalkine  MCP-1  MIP-1 α  IL-17 | F(1,24) = 35.83; p < 0.001  F(1,24) = 121.2; p < 0.001  F(1,24) = 32.74; p < 0.001 | F(1,24) = 6.412; p < 0.05  F(1,24) = 30.11; p < 0.001  F(1,24) = 6.193; p < 0.05  F(1,24) = 9.315; p < 0.01  F(1,24) = 4.532; p < 0.05 | | F(1,24) = 6.878; p < 0.05 | F(1,24) = 4.371; p < 0.05 | |

**Table S4.** Summary table of the two-way ANOVA of western blot, immunohistochemistry and electron microscopy data (only statistically significant data is shown).

Western blot data

| **P0 cortex** | Treatment | Genotype | Interaction |
| --- | --- | --- | --- |
| PLP  MBP  CD11b  MHCII  Caspase-3 | F(1,28) = 12.74; p < 0.01  F(1,28) = 6.481; p < 0.05  F(1,28) = 11.910; p < 0.01  F(1,28) = 7.205; p < 0.01  F(1,28) = 11.780; p < 0.01 | F(1,28) = 5.366; p < 0.05  F(1,28) = 18.920; p < 0.001  F(1,28) = 7.317; p < 0.05 | F(1,28) = 5.366; p < 0.05  F(1,28) = 18.920; p < 0.001  F(1,28) = 7.317; p < 0.05 |
| **P20 cortex** | Treatment | Genotype | Interaction |
| PLP  MBP  Synaptotagmin  Synapsin-IIa  Tuj-1  CD11b  MHCII  Caspase-3 | F(1,28) = 14.180; p < 0.001  F(1,28) = 10.280; p < 0.01  F(1,28) = 9.315; p < 0.01  F(1,28) = 5.646; p < 0.05 | F(1,28) = 10.54; p < 0.01  F(1,28) = 5.873; p < 0.05  F(1,28) = 7.902; p < 0.01  F(1,28) = 6.944; p < 0.05  F(1,28) = 4.929; p < 0.05 | F(1,28) = 10.654; p < 0.01  F(1,28) = 5.873; p < 0.05  F(1,28) = 7.902; p < 0.01  F(1,28) = 6.944; p < 0.05  F(1,28) = 4.929; p < 0.05 |
| **P66 cortex** | Treatment | Genotype | Interaction |
| PLP  MBP  Synaptotagmin  Synapsin-IIa  Tuj-1  CD11b | F(1,28) = 26.611; p < 0.001  F(1,28) = 7.646; p < 0.05  F(1,28) = 5.293; p < 0.05  F(1,28) = 6.419; p < 0.05  F(1,28) = 9.566; p < 0.01  F(1,28) = 9.169; p < 0.01 | F(1,28) = 7.426; p < 0.05  F(1,28) = 4.566; p < 0.05 | F(1,28) = 7.426; p < 0.05 |

**Immunohistochemistry** data

| **P20 cortex** | Treatment | Genotype | Interaction |
| --- | --- | --- | --- |
| MBP  Iba-1 | F(1,124) = 5.669 ; p < 0.05 | F(1,124) = 4.934 ; p < 0.05 | F(1,124) = 4.934 ; p < 0.05 |

**Electron microscopy** data

| **P20 cortex** | Treatment | Genotype | Interaction |
| --- | --- | --- | --- |
| Vesicle number  Postsynaptic density thickness | [F(1,12) = 41.96; p < 0.001]  [F(1,12) = 19.213; p < 0.001] | [F(1,12) = 34.102; p < 0.001] | [F(1,12) = 27.430; p < 0.001]  [F(1,12) = 21.261; p < 0.001] |
