# Supplementary material for: TLR4 response mediates ethanol-induced neurodevelopment alterations in a model of fetal alcohol spectrum disorders
Source: J Neuroinflammation. 2017 Jul 24;14:145. doi: 10.1186/s12974-017-0918-2 (PMC5525270; doi:10.1186/s12974-017-0918-2)
Supplement: Supplementary file 1 — Figure S1. Role of TLR4 in the expression of cytokines (IL-1β, IL-17) and chemokines (fractalkine, MCP-1, MIP-1α) in the cerebral cortices of the WT and TLR4-KO male pups on PND 0 and 20 exposed, or not, to ethanol during the embryonic and postnatal periods. PPEE: prenatal and postnatal ethanol exposure. Data represent mean ± SEM, n = 4 mice/group. *p < 0.05, compared to their respective control group. Table S1. Basal level of the brain proteins analyzed by western blotting between untreated or control (C) WT and TLR4-KO pups of PND 0 and PND 66. (ZIP 429 kb) [file 12974_2017_918_MOESM1_ESM.zip › Supplementary data 1.doc]

**Additional file 1**

**Figure S1:** Role of TLR4 in the expression of cytokines (IL-1β, IL-17) and chemokines (fractalkine, MCP-1, MIP-1α) in the cerebral cortices of the WT and TLR4-KO male pups on PND 0 and 20 exposed, or not, to ethanol during the embryonic and postnatal periods. PPEE: prenatal and postnatal ethanol exposure. Data represent mean ± SEM, n = 4 mice/group. * p < 0.05, compared to their respective control group.

**Table S1.** Basal level of the brain proteins analyzed by Western blotting between untreated or control (C) WT and TLR4-KO pups of PND 0 and PND 66.

|  |  | PND 0 | | PND 66 | |  |
| --- | --- | --- | --- | --- | --- | --- |
|  |  | Mean ± SEM | p | Mean ± SEM | p | |
| Caspase 3  (17 kDa) | WT C  TLR4-KO C | 1.00 ± 0.10  1.13 ± 0.18 | 0.53 |  |  | |
| CD11b | WT C  TLR4-KO C | 1.00 ± 0.04  0.96 ± 0.10 | 0.62 | 1.00 ±0.23  1.14 ± 0.12 | 0.66 | |
| MBP | WT C  TLR4-KO C | 1.00 ± 0.04  0.98 ± 0.06 | 0.91 | 1.00 ± 0.06  1.02 ± 0.14 | 0.89 | |
| MHC-II | WT C  TLR4-KO C | 1.00 ± 0.29  0.82 ± 0.30 | 0.69 |  |  | |
| PLP | WT C  TLR4-KO C | 1.00 ± 0.13  0.91 ± 0.20 | 0.71 | 1.00 ± 0.07  1.05 ± 0.17 | 0.76 | |
| Synapsin IIA | WT C  TLR4-KO C |  |  | 1.00 ± 0.10  1.16 ± 0.10 | 0.28 | |
| Synaptotagmin | WT C  TLR4-KO C |  |  | 1.00 ± 0.07  0.95 ± 0.11 | 0.71 | |
| Tuj-1 | WT C  TLR4-KO C |  |  | 1.00 ± 0.05  0.89 ± 0.13 | 0.42 | |

Statistical significance for the biochemical studies was determined by a Student’s t-test.
